# Feasible techniques in robotic thoracoscopic repair of congenital esophageal atresia: case report and literature review

**DOI:** 10.1186/s40792-021-01229-0

**Published:** 2021-06-15

**Authors:** Shuai Li, Guoqing Cao, Rongchao Zhou, Xi Zhang, Ying Zhou, Shao-tao Tang

**Affiliations:** 1 Department of Pediatric Surgery, Union Hospital, Tongji Medical College, Huazhong University of Science and Technology, 1277 Jie Fang Avenue, Wuhan, 430022 China; 2 Operation Room, Union Hospital, Tongji Medical College, Huazhong University of Science and Technology, Wuhan, 430022 China

**Keywords:** Esophageal atresia, Robotic surgery, Neonate

## Abstract

**Background:**

Robotic repair for esophageal atresia (EA) using da Vinci system is challenging. Specific surgical techniques need to be explored to overcome the current hurdles.

**Case presentation:**

Two cases with EA (type I and type III by Gross classification, respectively) were repaired using da Vinci robotic system. Step trocar insertion and asymmetric ports distribution techniques were used. The mean weight was 3.2 kg. Operative times were 95 min totally, with the anastomotic time of 27.5 min. Follow-up duration was 12 months. Esophageal fistula reoccurred in one case. None was confirmed anastomotic stricture.

**Conclusion:**

Step trocar insertion procedure and asymmetric ports distribution technique are effective in robotic esophageal atresia.

## Highlights

Robot surgery system has unique advantages in the narrow space, but its application in neonatal surgery is still controversial and challenging. Trocar insertion into the narrow intercostal space and prevention to decrease instruments collision were the current main hurdles.

As the first report on the procedure details of robotic esophageal atresia surgery, the Step procedure and the asymmetric ports distribution technique perfectly solved these above problems.

## Background

Robotic-assisted surgery is still debated, but is already having a role in pediatric minimal invasive surgery, even in neonates. Meehan et al. initiated a study focused on small children, which presented 45 patients of less than 10 kg who underwent robotic surgery. The smallest reported patient weighed 2.2 kg in this series. Though one EA repair was pioneer attempted, it was demonstrated that limited thoracic domain could get irreparable collided when the patient’s weight was below 4 kg [1]. Followed this, Quentin Ballouhey reported three of these neonatal patients with EA. Two procedures were converted to thoracotomy for internal cluttering. The main cause was the reduced working space and difficulty in trocar insertion. Fortunately, no perioperative complications or mechanical malfunctions with the robots and no peri- or postoperative severe hypercapnia or acidosis were observed [2].

However, EA is a congenital anomaly and should be repaired in neonatal period when weighing lower than or around 3 kg. Other than the above two reports, no successful technical detail for robotic thoracoscopic EA repair was available to our knowledge. Herein, we present our preliminary experience in robotic repair of esophageal atresia and the technical details to overcome the above hurdles.

## Case report

### General information

Case 1 was a full-term born boy with a weight of 2.8 kg and diagnosed as type I EA 7 days after. He was transferred from NICU weighing 3.1 kg on the 15th day after birth. System screening before operation revealed nothing except small atrial septal defect (3 mm). Robotic repair for esophageal atresia was performed after pneumonia was controlled and hypoproteinemia was corrected. The gap between proximal and distal ends was 1.8 cm. Operative times were 95 min totally, with the docking time of 20 min and the anastomotic time of 30 min. No perioperative severe hypercapnia or acidosis was not observed. The body temperature was slightly high in the first three postoperative days (not higher than 37.9 ℃), and returned to normal thereafter. No surgical site infection occurred. The patient was extubated 24 h after operation. Feeding through nasogastric tube was administrated on 3 days after operation and orally according to the result of contrast X-ray done on 7 days after operation. This case was followed up for 19 months and no symptomatic anastomotic stricture occurred.

Case 2 was also a full-term born boy with a weight of 3.1 kg and diagnosed as type III EA 2 days after. He was transferred from NICU weighing 3.3 kg on the 7th day after birth. System screening before operation revealed nothing. Robotic repair for esophageal atresia was performed successfully. The gap between proximal and distal ends was 2.2 cm. Operative times were 90 min totally, with the docking time of 15 min and the anastomotic time of 27 min. No perioperative severe hypercapnia or acidosis was observed. The body temperature was not higher than 38℃ in the first three postoperative days. No surgical site infection occurred. The patient was extubated 72 h after operation. Feeding through nasogastric tube was administrated on 3 days after operation. He got dyspnea on the 10th day after operation for aspiration pneumonia and recovered by noninvasive ventilator-assisted breathing and antibiotics therapy. The contrast X-ray was done on 22th day after operation and no fistula was found. He fed well orally and then was discharged. Unfortunately, he got refistulized 6 weeks after the initial procedure and was repaired under thoracotomy. This case was followed up for 5 months and no symptomatic anastomotic stricture occurred.

### Technique

**Fig. 1:**
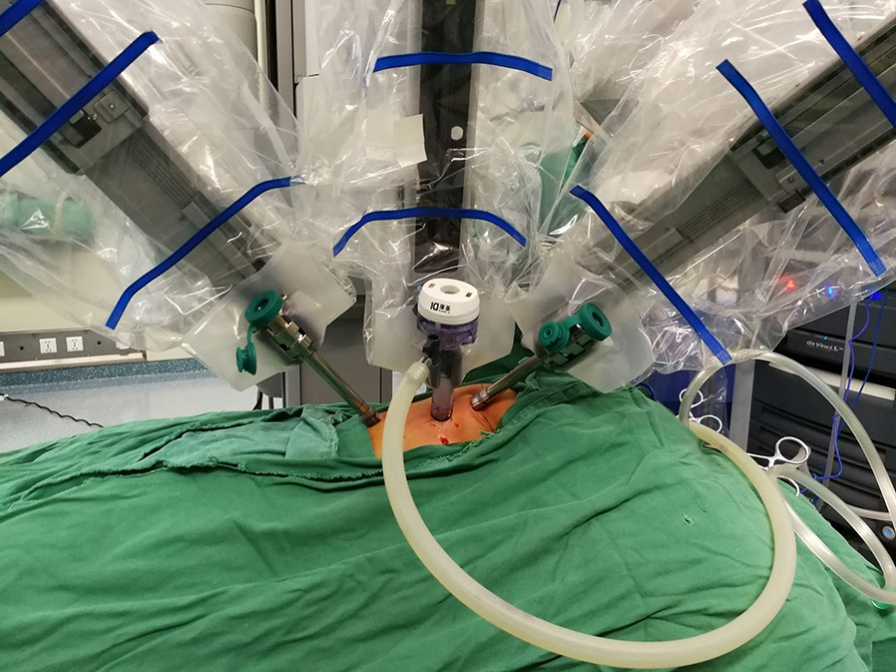
The robotic surgical cart positioned at the front of the patient

**Fig. 2:**
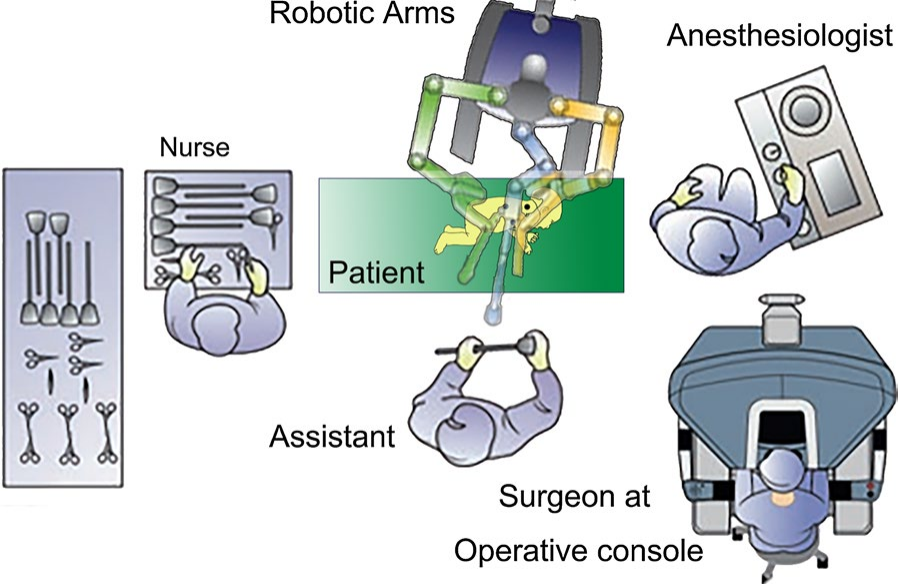
The relative position of functional parts in the robotic surgery room

**Fig. 3:**
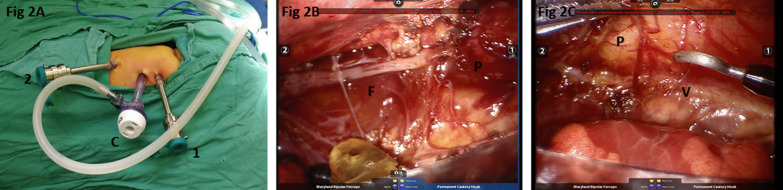
Illustration of the operation. **A** The positions of the ports: C, camera port; 1, 2, instruments ports. **B** F, the fibrous cord; P, the proximal pouch of the esophagus. **C** V, the vagus nerve

After routine general anesthesia, the patient was positioned in a left lateral decubitus position (30° prone). The robotic surgical cart (da Vinci Si; Intuitive Surgical, Inc, Sunnyvale, Calif) was positioned at the front of the patient (Fig. 1). The relative position of several functional parts in the robotic surgery room is shown in Fig. 2. Three-arm Standard da Vinci Surgical Robot with one camera arm and two instrument arms was utilized. CO_2_ was insufflated at a pressure of 6 mm Hg with a flow of 1 L/min. The camera port (12 mm) was positioned in the fifth intercostal space at the posterior axillary line. The other two ports (8 mm) were placed in the third and the seventh intercostal space at the anterior axillary line, respectively, in asymmetric fashion (Fig. 3A). These three ports were inserted into the thoracic cavity using Step technique: firstly, a 3-mm trocar was inserted at the middle of the intercostal space to form the initial channel; the channel was gradually enlarged using a 5-mm trocar and 8-mm trocar to a 12 mm target size. Associated port (3 mm) for assisted instruments was placed at the sixth intercostal space of anterior axillary line.

**Fig. 4:**
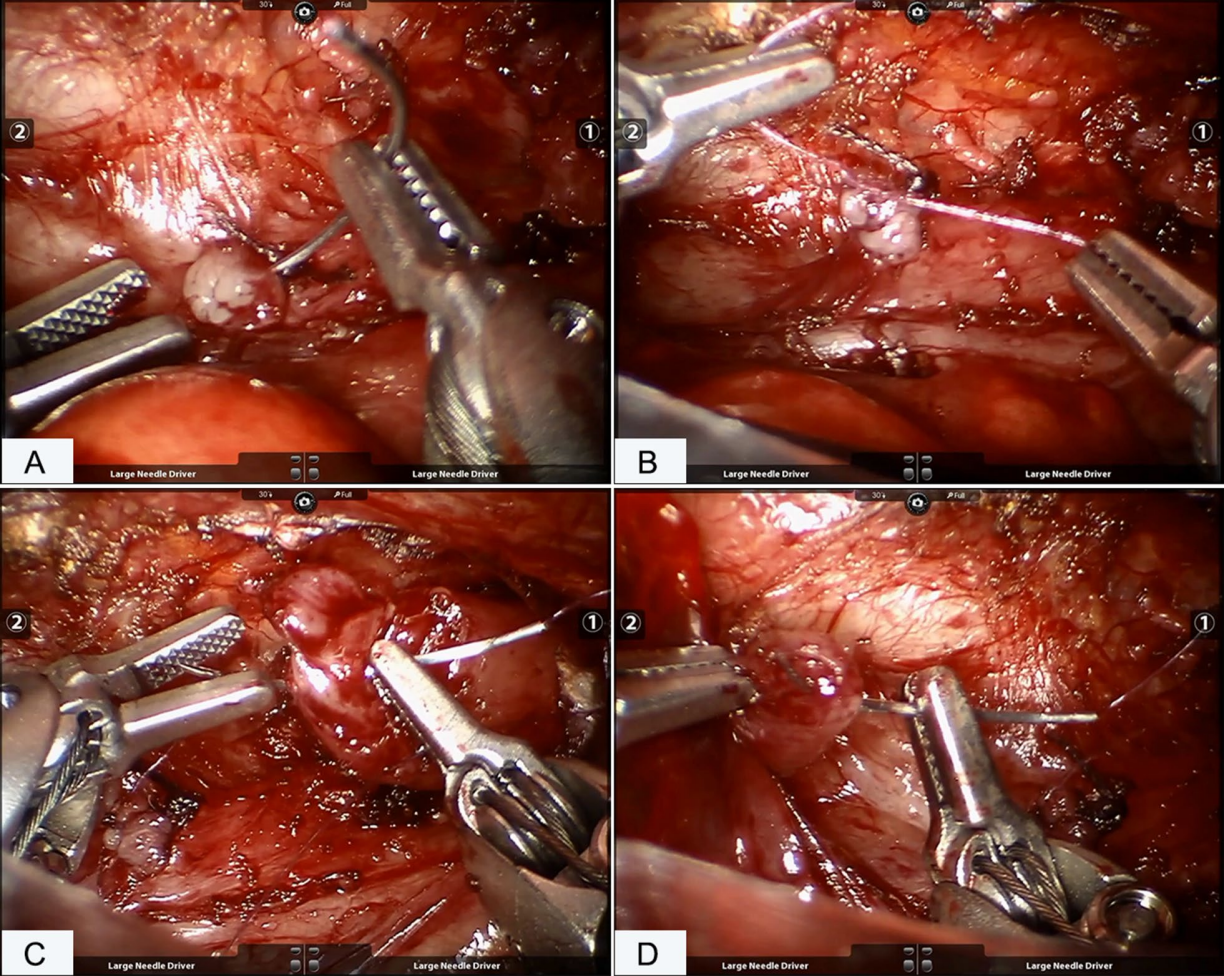
The fistula closure and esophageal anastomosis. **A** suturing the fistula; **B** making knots and closing the fistula; **C** suturing the proximal pouch of the esophagus; **D** suturing the distal end of the esophagus

The dissection was performed using a Maryland grasper in the left robotic arm and a hook-tip cautery in the right robotic arm. The azygos was identified and mobilized for a short segment and then cauterized and divided. With the assistance of the nasogastric tube, the upper pouch was identified and dissected out. After the upper pouch was mobilized, the fibrous cord connected with it was retracted (Fig. 3B), making it easy to visualize the distal segment. The fistula was mobilized and closed proximally with double figure 8 sutures (Fig. 4A, B). The vagus nerve accompanying the esophagus was clearly displayed and protected (Fig. 3C) during the whole procedure. Once adequate mobilization was achieved, the atretic ends of the proximal (Fig. 4C) and distal (Fig. 4D) segments were resected and then the anastomosis was performed using a 5-0 absorbable suture in an interrupted fashion. The first suture was done with the knots intraluminal with additional five sutures knotting extraluminally on the back wall. The NG tube was then passed under direct vision into the lower pouch after the back half of the anastomosis. The anastomosis of the anterior wall was the completed by another six sutures with the NG tube ensuring patency of the esophagus. A chest tube was placed through the lower trocar site.

## Discussion

Despite numerous advantages, thoracoscopic esophageal anastomosis is challenging. The spatial constraints of the thoracic cavity combined with surgeon tremor, large translated motions at the instrument tips, and a decreased view of the operative field increased not only the risk of injury to surrounding organs, but also the difficulty of suturing and knot-tying tasks [3]. In addition, the location of the lesion itself and the thoracoscopic rigid equipment made the operations not in accordance with ergonomic considerations, and finally made the surgeon extremely exhausted.

Theoretically, robotic surgery system possesses superior techniques and friendly design concept over standard thoracoscopic rigid instruments. The articulating instruments with 7 degrees of freedom permit suturing in locations that are difficult to reach, and make dissection around the surgical target at adequate angles. In addition, 3-dimension camera is controlled by the surgeon oneself and internal articulation of the instruments allows the surgeon eye–hand integration to perform flexible and precise maneuvers comfortably [4]. Moreover, stable view and tremor filtration technique is especially helpful for esophageal anastomosis.

However, additional hurdles would come when current robot surgery system was applied: unavoidable instruments collision and inserting huge trocars through the tiny intercostal space. Initially, the minimum distance required between ports is 8 cm and 5–6 cm for later generation robots [5]. Previously, patient’s weight below 4 kg could get irreparable collided for thoracic robotic procedures [1]. According to our experience, in neonates weighing over 3 kg, the width of the hemithorax is only around 7 cm, roughly meeting the requirement. Since the distance between ports cannot reach that requirement, we developed an asymmetric ports distribution technique (Fig. 3A): the distance between the right and the camera ports was only 3 cm, while the distance between the left and the camera was 5 cm. During operation, the instruments remoted by the right hand only maneuvered with the inner-articulating part to avoid robotic arms collisions outside. The motion on the narrow side (right side) was slightly restricted sometimes, but no severe external arms collisions took place. To get enough freedom, the requisite internal length of the instruments is 5.61 cm to keep the articulating instruments functional [2]. Additional solution was that placing the trocars outside of the patient after instruments introduction to provide the instruments an extra 1.5–2.0 cm of maneuverability [1].

Obviously, it was difficult to insert 12 mm and 8 mm trocars into the intercostal space in neonates [2]. It is possible for the huge trocars to cause rib fracture. We applied a Step procedure firstly used in cannula making in anorectal plasty for high ARM [6]. By dilating the intercostal space gradually, the huge trocars were place on the chest wall successfully. None case got real fracture or postoperative thoracic deformity.

It is acknowledged that the convincingness of this study is limited for series size (only 2 cases). Efforts are still needed to summarize global cases in perspective manner using the next-generation robotic surgery system. As Cundy reviewed, robotic surgery is an expanding and diffusing innovation in pediatric surgery [7]. Taking advantage of the robotic surgery system and finding ways to overcome obstacles can benefit patients finally.

To conclude, Step procedure and asymmetric ports distribution were effective in neonatal thoracic robotic procedures. Stable view and tremor filtration made the esophageal anastomosis more concise. However, there were constant complaints regarding the size of the instruments and trocars.

## Data Availability

Available and could be obtained by asking the corresponding author for specific information.

## Abbreviations

EA: Esophageal atresia
NICU: Neonatal intensive care unit
NG tube: Nasogastric tube
ARM: Anorectal malformation
